# Adrenal Venous Sampling in Primary Aldosteronism: The Usefulness of Contralateral Suppression Index

**DOI:** 10.1155/2019/1604367

**Published:** 2019-09-03

**Authors:** Natalia Treistman, Aline Barbosa Moraes, Stéphanie Cozzolino, Patrícia de Fatima dos Santos Teixeira, Leonardo Vieira Neto

**Affiliations:** Department of Internal Medicine and Endocrine Unit, Medical School and Clementino Fraga Filho University Hospital—Federal University of Rio de Janeiro, Rio de Janeiro, Brazil

## Abstract

Adrenal venous sampling (AVS) is the gold standard test to differentiate the unilateral from the bilateral form in patients with primary aldosteronism (PA) although it may be a difficult procedure, especially the successful cannulation of the right adrenal vein. In this report, we describe a 49-year-old female patient diagnosed with PA, after investigating resistant hypertension and refractory hypokalemia. Abdominal computed tomography scan revealed a 2.5 cm adenoma on the right adrenal vein. AVS was performed under cosyntropin infusion. Aldosterone and cortisol concentrations were obtained from the right and left adrenal veins and inferior vena cava (IVC). Cortisol on each adrenal vein divided by cortisol on IVC confirmed successful cannulation of the left side only, which makes it impossible to calculate the lateralization index (LI). From the data on the left adrenal vein and IVC, the aldosterone-to-cortisol ratio divided by the IVC aldosterone-to-cortisol ratio was less than 1.0, suggesting that the left adrenal vein was suppressed with the excess aldosterone originating from the contralateral side (contralateral suppression index (CSI)). Right adrenalectomy was performed; postoperative hypoaldosteronism was confirmed. This report highlights the importance of CSI obtained in AVS when technical difficulties occur making it impossible to obtain LI, which is most commonly used to decide between surgical and clinical management of PA.

## 1. Introduction

Primary aldosteronism (PA), with its prevalence reaching 20% of patients with resistant hypertension, is the most common curable form of secondary hypertension in patients referred to specialized centers [1]. The two most common causes of PA are idiopathic aldosteronism (IHA), which is best treated with an aldosterone antagonist, and unilateral aldosterone-producing adenoma (APA), which can be potentially cured by surgery. However, differentiation between these two diseases remains challenging, given that reliance on adrenal images only can lead to about 37.8% of inaccurate results [2].

Adrenal venous sampling (AVS) remains the gold standard test to differentiate between the most common causes of PA. However, it is a technically demanding procedure, which has a high rate of failure even in centers with experts, leading to about 69.5% of unsuccessful or unilaterally selective exams in a German report [3]. Although several protocols have been studied to improve the accuracy of catheterization, interpretation of results with contralateral suppression index (CSI) remains debatable.

Our case describes a patient whose diagnosis of unilateral APA was made through the presence of contralateral suppression criteria on AVS results.

## 2. Case Presentation

A 49-year-old female patient was referred to our Endocrinology Service for investigation of resistant hypertension, which had been present since she was 33 years of age, and persistent hypokalemia. She was on clonidine (0.3 mg/day), hydralazine (150 mg/day), methyldopa (450 mg/day), and diltiazem (180 mg/day). Her medical background included a history of chronic kidney disease (CKD) stage IV and family history of hypertension. Her physical examination was normal, except for high blood pressure (190 × 130 mmHg) and an overweight body mass index (BMI) (26 kg/m^2^).

A 24-hour ambulatory blood pressure monitoring confirmed resistant hypertension, despite treatment. On further investigation, renal imaging by ultrasound revealed loss of corticomedullary differentiation; her echocardiogram revealed left ventricular hypertrophy.

The patient underwent an evaluation for secondary hypertension, excluding renovascular disease assessed by eco-Doppler and dynamic renal scintigraphy with 99mTc-DTPA (diethylene triamine-pentaacetic acid labeled with 99mTc).

**Figure 1:**
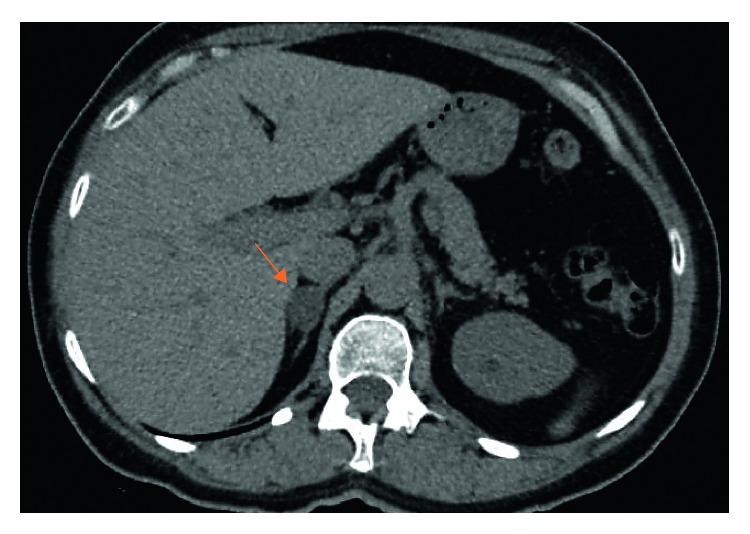
Abdominal computed tomography (CT) scan showing a 2.5 cm adrenal nodule suggestive of adenoma on the right (arrow).

A laboratory evaluation revealed normal levels of sodium (142 mEq/L; reference range (RR): 136–145 mEq/L) and hypokalemia (3.3 mEq/L; RR: 3.5–5.1 mEq/L) despite the intake of over 4 g of potassium per day, undetectable plasma renin activity (PRA <0.2 ng/mL/h, RR: 0.2–2.8 ng/mL/h), and inappropriately high plasma aldosterone concentration (PAC) for PRA (PAC: 14.2 ng/dL, RR: 2.5–31.5 ng/dL). The aldosterone-to-renin ratio (ARR) was clearly high, at least 71. The estimated glomerular filtration rate (GFR) was 29 mL/min/1.73 m^2^ by the Modification of Diet in Renal Disease (MDRD) formula. A 2.5 cm nodule on the right adrenal, suggestive of adenoma (Figure 1), was detected on abdominal computed tomography (CT) scan.

The patient underwent AVS using continuous cosyntropin stimulation. The infusion of 50 mcg/hour was initiated 30 minutes before and continued throughout the procedure. The initial access was through both femoral veins with the intention of performing simultaneous blood sampling. However, due to technical difficulties, the blood samples were obtained sequentially under fluoroscopic guidance. A report on the procedure described difficulty in obtaining the sample from the right adrenal vein due to its small caliber, which led to sampling from the vein's ostium. Peripheral samples for aldosterone and cortisol were also obtained from the inferior vena cava (IVC). Blood samples were labeled and sent for analysis of cortisol and aldosterone (Table 1). As expected, unsuccessful cannulation of the right adrenal vein, due to its very small caliber, was confirmed by a very low selectivity index (SI). However, successful cannulation was performed on the left adrenal vein, as confirmed by a high SI, and they revealed contralateral suppression of the left adrenal gland, assessed by CSI (Table 1).

The patient underwent a laparoscopic right adrenalectomy, and the histopathological examination confirmed an adrenocortical adenoma. One month after surgery, postural hypotension was confirmed, and the patient was administered as losartan, hydralazine, and clonidine. Then, the doses of medicines were progressively reduced and completely withdrawn by the 45^th^ postoperative day. The patient remained normotensive and eukalemic in subsequent evaluations. After 3 months, the patient presented with orthostatic hypotension and hyperkalemia; renal function progressively worsened (GFR 22 mL/min/1.73 m^2^ by MDRD). Clinical suspicion of postoperative hypoaldosteronism was raised, and she was later administered with 0.05 mg/day of fludrocortisone. Afterwards, serum potassium returned to its normal range, and blood pressure control improved after 15 days, but evolution of renal injury persisted (GFR: 16 mL/min/1.73 m^2^), which was probably secondary to previous development of renal microvascular disease.

## 3. Discussion

Diagnosis of PA is important and should be pursued when dealing with a patient with resistant hypertension since it is potentially curable. The use of ARR is well established as the most reliable screening method for PA, and in case settings, such as the one described (spontaneous hypokalemia with suppressed plasma renin levels and elevated aldosterone), there is no need for further confirmatory testing [4].

When proceeding to adrenal imaging after confirmation of PA diagnosis, it is important to take into account the patient's age. It has been reported that the prevalence of nonfunctioning adrenal adenomas increases with age and so it is an important cause of false positives [5]. Therefore, we chose to perform AVS to differentiate between the two possible diagnoses, IHA and APA.

AVS remains the gold standard test to differentiate between IHA and APA in cases of PA; many experts advocate that it should be offered to all patients before surgery [6]. However, it is a technically difficult procedure even among experienced interventional radiologists; even the best centers have a high rate of suboptimal exams [3]. This technical AVS-related difficulty is due to the anatomy of the right adrenal vein, that is, its small caliber and acute angle of flow into the IVC [6]. Adequate catheter position may be confirmed by using the adrenal vein to IVC cortisol ratio (selectivity index (SI)). With the continuous cosyntropin infusion protocol, the SI is typically more than 5 : 1. For this case, successful catheterization was confirmed on the left side only (Table 1). Failure to catheterize this vein was reported to be about 59% in a multicenter study [3].

When faced with such difficulty, interpretation of the AVS results reaches an impasse since it is quite impossible to calculate the lateralization index (LI), which depends on the ratio of aldosterone-to-cortisol of both sides. The LI is paramount and used in centers that perform AVS to decide between medical and surgical management [3, 4, 6]. Since the right adrenal vein was not successfully catheterized in the present study, LI was impossible to calculate.

This dilemma has led to the need to create a parameter to be used on unilaterally selective exams, when bilateral results are not available. The use of a unilateral study design is based on the assumption that if the patient has unequivocal biochemical evidence of PA and one side is suppressed, then the excess aldosterone should come from the contralateral side. This may allow diagnosis even when the right adrenal vein cannot be successfully catheterized.

The formula used to calculate CSI is generally accepted to be the ratio of aldosterone-to-cortisol of the contralateral adrenal gland, divided by the same ratio of the IVC. In our patient, the contralateral adrenal gland corresponded to the adequately catheterized side (left adrenal vein). Concerning the cutoff values when calculating the CSI, no consensus has been reached in literature. It varies greatly, from less than 1.4 to less than 0.5, though more than 1 author uses at least 1.0 as the cutoff [7–9]. Our patient presented with a CSI of 0.273, which indicates suppression of aldosterone secretion by the noninvolved adrenal gland. This parameter has been reported to be a predictor of outcomes, such as blood pressure improvement in patients which led to adrenalectomy due to PA [8].

A disagreement as to whether CSI may be a useful parameter to determine blood pressure outcomes in patients scheduled for surgery for correction of PA exists, with some authors advocating for it and others showing evidence that its use does not correlate to blood pressure improvement [7–9]. Our patient became normotensive and eukalemic after adrenalectomy, without need for drugs to control blood pressure or potassium replacement.

Notwithstanding the lack of consensus, a multicenter study has shown that most (12 of 20) centers use data of unilaterally selective studies when bilateral results are unavailable [10].

## 4. Conclusion

This report brings to light the usefulness of the CSI parameter obtained in AVS when technical difficulties occur resulting in a difficulty to obtain other data, such as LI, which are most commonly used to decide between surgical and clinical management of primary hyperaldosteronism. It also advocates the use of CSI as a tool to predict blood pressure outcomes in PA patients.

## Figures and Tables

**Table 1:** Results of aldosterone and cortisol obtained from adrenal vein sampling under cosyntropin continuous stimulation.

Vein	Aldosterone (ng/dL)	Cortisol (*μ*g/dL)	Selectivity index	Contralateral suppression index
IVC	30.5	34.0	—	—
Right AV	39.0	32.6	0.95	NA^*∗*^
Left AV	47.3	193.0	5.67	0.273

A, aldosterone; AV, adrenal vein; C, cortisol; IVC, inferior vena cava; IV, right iliac vena; NA, not applicable. Selectivity index: cortisol on adrenal vein divided by cortisol on inferior vena cava. A ratio of at least 5 : 1 is needed for one to be confident that the adrenal veins were successfully catheterized. Thus, only the left side was adequately sampled (selectivity index of 5.67). Contralateral suppression index: aldosterone/cortisol on the adequately catheterized side divided by aldosterone/cortisol on the inferior vena cava. The contralateral aldosterone-to-cortisol ratio (0.245) is less than the IVC aldosterone-to-cortisol ratio (0.897), with a ratio less than 1.0 (0.273 in this case), which is indicative of suppression of aldosterone secretion by the noninvolved adrenal gland. ^*∗*^It is not applicable because the right adrenal vein was not successfully catheterized.
